# Motor Skills and Exercise Capacity Are Associated with Objective Measures of Cognitive Functions and Academic Performance in Preadolescent Children

**DOI:** 10.1371/journal.pone.0161960

**Published:** 2016-08-25

**Authors:** Svend Sparre Geertsen, Richard Thomas, Malte Nejst Larsen, Ida Marie Dahn, Josefine Needham Andersen, Matilde Krause-Jensen, Vibeke Korup, Claus Malta Nielsen, Jacob Wienecke, Christian Ritz, Peter Krustrup, Jesper Lundbye-Jensen

**Affiliations:** 1 Department of Nutrition, Exercise and Sports, University of Copenhagen, Copenhagen, Denmark; 2 Department of Neuroscience and Pharmacology, University of Copenhagen, Copenhagen, Denmark; 3 Copenhagen Centre for Team Sport and Health, University of Copenhagen, Copenhagen, Denmark; 4 Frederikssund Municipality, Frederikssund, Denmark; 5 Sport and Health Sciences, College of Life and Environmental Sciences, University of Exeter, Exeter, United Kingdom; Pondicherry Institute of Medical Sciences, INDIA

## Abstract

**Objective:**

To investigate associations between motor skills, exercise capacity and cognitive functions, and evaluate how they correlate to academic performance in mathematics and reading comprehension using standardised, objective tests.

**Methods:**

This cross-sectional study included 423 Danish children (age: 9.29±0.35 years, 209 girls). Fine and gross motor skills were evaluated in a visuomotor accuracy-tracking task, and a whole-body coordination task, respectively. Exercise capacity was estimated from the Yo-Yo intermittent recovery level 1 children's test (YYIR1C). Selected tests from the Cambridge Neuropsychological Test Automated Battery (CANTAB) were used to assess different domains of cognitive functions, including sustained attention, spatial working memory, episodic and semantic memory, and processing speed. Linear mixed-effects models were used to investigate associations between these measures and the relationship with standard tests of academic performance in mathematics and reading comprehension.

**Results:**

Both fine and gross motor skills were associated with better performance in all five tested cognitive domains (all P<0.001), whereas exercise capacity was only associated with better sustained attention (P<0.046) and spatial working memory (P<0.038). Fine and gross motor skills (all P<0.001), exercise capacity and cognitive functions such as working memory, episodic memory, sustained attention and processing speed were all associated with better performance in mathematics and reading comprehension.

**Conclusions:**

The data demonstrate that fine and gross motor skills are positively correlated with several aspects of cognitive functions and with academic performance in both mathematics and reading comprehension. Moreover, exercise capacity was associated with academic performance and performance in some cognitive domains. Future interventions should investigate associations between changes in motor skills, exercise capacity, cognitive functions, and academic performance to elucidate the causality of these associations.

## Introduction

The association between physical activity, cognition, and academic achievement in children has received great focus in recent years [1–5]. Several studies have shown that higher-fit children tend to perform better than lower-fit children in cognitive tests [2, 4, 6–8], and other studies have found a weak, but positive relationship between measures of aerobic capacity and academic achievement in children [3, 4, 9–12].

Whereas previous studies have found positive associations between aerobic fitness and performance in cognitive or academic tests, recent studies have also documented positive associations between motor functions and academic achievement. These findings underpin a potential positive role of physical activity in children and indicate that development of motor skills is positively related to scholastic learning. While studies on aerobic fitness in children found positive associations with performance in cognitive tests and academic performance, the relationship between motor skills and cognitive functions in schoolchildren remains however less well investigated. To our knowledge no study has previously focused on simultaneous assessment of motor functions, exercise capacity, cognitive functions and academic performance in the same cohort of children and it is the aim of the present study to a) investigate the potential associations between motor skills and cognitive functions and b) to detail the knowledge on associations between motor skills, exercise capacity, cognitive functions and academic performance in preadolescent children.

### Relationship between Motor Skills and Cognitive Functions

We often consider the development of motor skills as separate from cognitive development, and the terminology itself clearly separates these functions. However, motor and cognitive development may be fundamentally interrelated. In order for cognitive processes to have functional implications, these must influence and be influenced by our actions and thus engage the motor system and perceptual functions. Conversely, cognitive processes may assist decision-making, motor control and motor skill learning processes. Cognitive and motor functions display equally protracted time courses during development, and in the event that cognitive development is perturbed (e.g. by neurological disorders), motor development is often also adversely affected [13]. In recent years, it has been demonstrated that many tasks require parallel activation of cognitive-motor circuitries encompassing the prefrontal cortex, the striatum and the cerebellum, and not only can the prefrontal cortex influence motor control, but the cerebellum may also be important for cognitive functions [13].

Both fine and gross motor skills are highly important for our ability to manage and succeed in everyday life. According to conventional theory, motor skills engage procedural, non-declarative memory, but the mechanisms subserving formation and retention of declarative and non-declarative memory are to a large degree similar [14]. Although memory systems are distinct, recent studies have made it conceivable that declarative and procedural memories can, and do indeed, interact [15], and memories are not necessarily confined to independent systems. It is therefore a primary aim of the present study to determine whether fine and gross motor skills are associated with objective measures of cognitive functions in preadolescent children.

### Relationship between Motor Skills, Cognitive Functions and Academic Performance

During recent years studies have found positive associations between motor functions and academic performance in children. A large-sample study of more than 8,000 adolescents showed that self-reported physical activity and obesity mediated the association between parent-reported childhood motor function and academic achievement at age 16 [12]. Moreover, a recent 9-year intervention study involving daily physical education and motor practice showed that children with motor skill deficits performed worse in academic tests than children with no deficits. Importantly, this study also demonstrated that daily physical education (PE) and motor skill training during the compulsory school years not only led to improved motor skills but was also accompanied by improved school performance in adolescence [11].

The positive associations between motor functions and academic performance and the positive effects of long-term motor skill training and physical activity reported by Ericsson & Karlsson (2014) are indeed interesting. We therefore set out to investigate the interrelationship between motor skills, objective measures of cognitive functions and academic performance. Solving mathematical problems requires cognitive functions such as working memory and sustained attention while reading comprehension draws on working memory and also requires accurate and fluent decoding of words, semantic memory, vocabulary knowledge, and reasoning skills. The association between performance in specific cognitive domains and academic performance is therefore also of fundamental interest.

In the present study, we expected to find a positive relationship between fine and gross motor skills and performance in specific cognitive domains. We further hypothesized that we would find a positive relationship between both performance in cognitive tests, fine and gross motor skills and academic performance. To the best of our knowledge, such associations have not been demonstrated previously using objective tests in preadolescent children. Finally, we expected to confirm the finding from previous studies that exercise capacity is positively related to performance in some cognitive domains and to academic performance.

## Materials and Methods

The initial sample comprised 423 (209 girls) of the 447 invited children in third grade, aged 9.29±0.35 years (range 8‒10 years), from 20 school classes at seven Danish municipal schools. Three schools were located in the suburb of the Frederikssund Municipality, while four schools were from the Danish capital, Copenhagen (Copenhagen Municipality).

The study was approved by the Committees on Biomedical Research Ethics for the Capital Region of Denmark (J.no. H-3-2013-038). Child assent and written informed parental consent were obtained for all participants prior to initiation of experimental procedures.

On four separate test days, the children participated in tests of motor skills, exercise capacity, anthropometric measures (body composition), performance in standardised cognitive tests, and academic performance (mathematics and reading comprehension), as well as a physical examination and an interview with a medical doctor specialised in general medicine who asked about lifestyle, participation in organised leisure sports, and determined their Tanner stage [16]. All tests were performed during week 3‒6 of the school year.

### Anthropometric Measures

An overview of the population included in the study can be found in Table 1. Height was measured to the nearest millimetre (235 Heightronic Digital Stadiometer, QuickMedical, Issaquah, WA, US) and the children were then weighed to the nearest 0.1 kg (Tanita WB-110MA, Tanita, Europe) while barefoot and wearing light clothing. Finally, whole-body composition was determined by Dual-energy X-ray absorptiometry (Lunar Prodigy; GE Medical Systems, Madison, Wisconsin, USA) using Encore software version 13.5 (Encore, Madison, USA). These measures were obtained to characterize the population included in the study.

**Table 1:** Characteristics of the study population and their overall test performance.

Variable	*n*	Boys (n = 214)	Girls (n = 209)	All (n = 423)
***Basic info***				
Age (years)	*422*	9.30 ± 0.36	9.29 ± 0.32	9.29 ± 0.35
Height (cm)	*418*	139.4 ± 5.6	137.5 ± 6.5	138.5 ± 6.1
Body mass (kg)	*418*	32.7 ± 5.6	32.5 ± 7.0	32.6 ± 6.3
BMI	*418*	16.7 ± 2.2	17.1 ± 2.7	16.9 ± 2.5
Body Fat (%)	*410*	19.6 ± 8.1	26.8 ± 8.9	22.7 ± 9.1
Tanner stage (1/2/3)	*419*	212 / 0 / 0	160 / 45 / 2	372 / 45 / 2
Organised leisure sports (y/n)	*420*	155 / 58	139 / 68	294 / 125
***Motor skills and exercise capacity***				
Fine motor skill (VAT score)	*411*	37.9 ± 14	37.1 ± 13.6	37.4 ± 13.8
Gross motor skill (motor-skill wall, s)	*417*	69.1 ± 13.9	69.5 ± 14.9	69.3 ± 14.4
Exercise capacity (YYIR1C, m)	*410*	844 ± 454	578 ± 335	713 ± 421
***Cognitive performance***				
Simple reaction time (ms)	*414*	332.3 ± 63.5	345.4 ± 67.1	338.7 ± 65.6
Sustained attention (RVP, #misses)	*414*	5.6 ± 3.2	6.2 ± 3.8	5.9 ± 3.5
Spatial working memory (SWM, #errors)	*414*	18.4 ± 8.4	15.7 ± 8.3	17.1 ± 8.4
Free-recall word memory (#words)	*413*	5.6 ± 1.8	6.2 ± 1.9	5.9 ± 1.9
Paired associates learning (PAL, #errors)	*414*	15.1 ± 13.7	12.6 ± 11.4	13.9 ± 12.6
***Academic performance***				
Mathematics (#correct)	*402*	33.9 ± 7.7	32.2 ± 8.5	33.0 ± 8.3
Reading comprehension (#correct)	*401*	41.5 ± 14.5	41.9 ± 14.3	41.7 ± 14.4

All data are presented as mean ± SD except for Tanner stage and physical activity, where the distributions of the scores and responses, respectively, are presented. Abbreviations: BMI, body mass index; YYIRC1, Yo-Yo intermittent recovery level 1 children’s test; VAT, visuomotor accuracy tracking; SWM, spatial working memory; RVP, rapid visual processing.

### Motor Skills and Exercise Capacity

#### Fine motor skill—visuomotor accuracy tracking (VAT)

**Fig 1:**
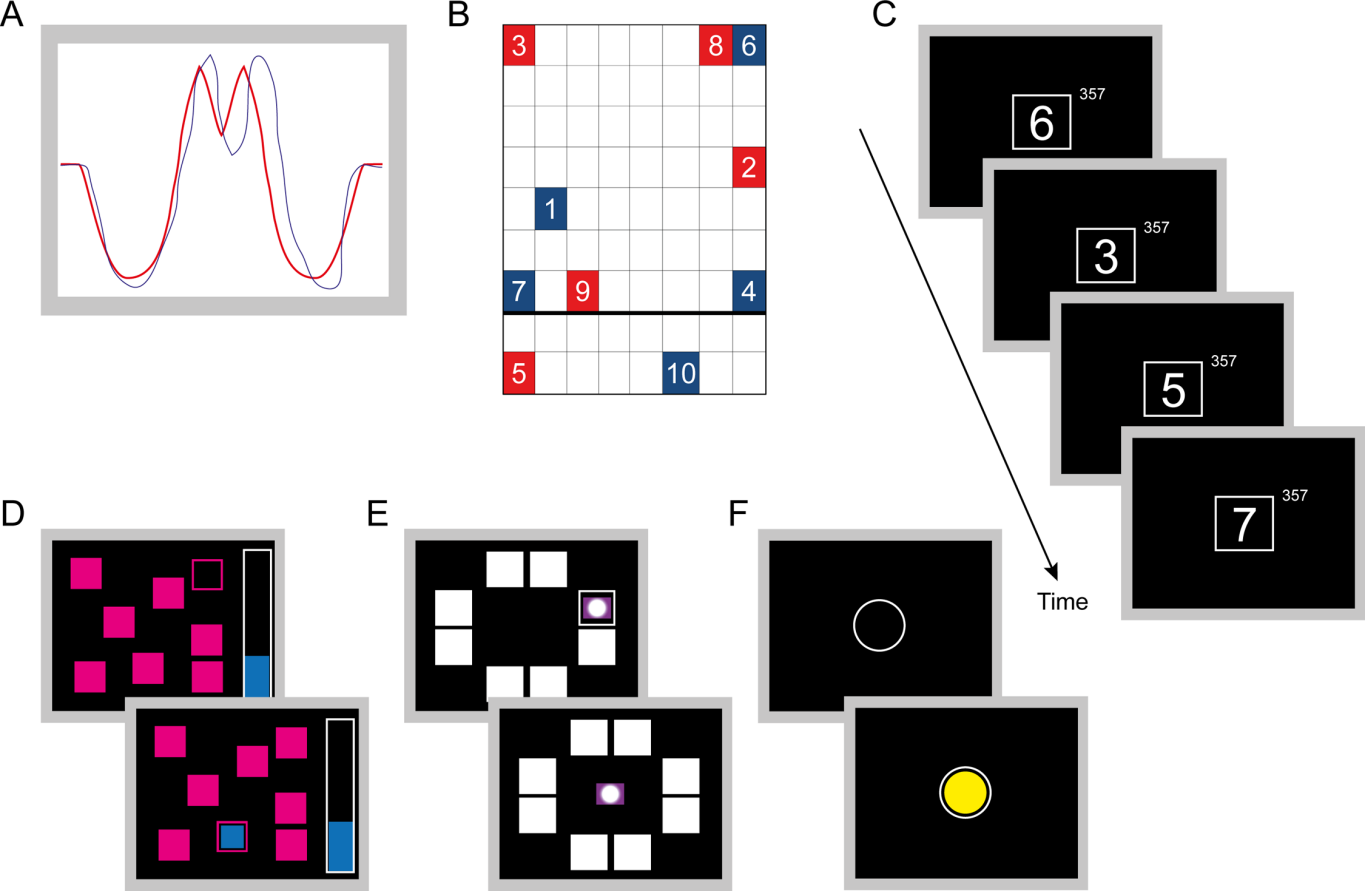
Motor skill and cognitive tests. (A) Fine motor skill–visuomotor accuracy tracking (VAT). The children were instructed to track, as accurately as possible, a fixed target (red line) by using a computer mouse to control the vertical position of a moving trace (blue line). (B) Gross motor skill–stage 3 of the coordination wall. The children had a red dot on the right hand and a blue on the left hand, and were instructed to touch the numbers from one to ten in the correct order with the hand (above the thick line) or foot (below) matching the colour of the number as fast as possible. (C) Rapid Visual Information Processing (RVP) measured sustained attention. A white box appeared in the centre of the computer screen, inside which digits, from 2 to 9, appeared in a pseudo-random order at the rate of 100 digits per minute. Children were requested to press a button on the press-pad when the target sequence (3-5-7) appeared. (D) Spatial Working Memory (SWM). The child had to search for a blue token hidden inside one of the coloured boxes shown on the screen. The number of boxes was gradually increased from three to eight. The outcome measure used here was total number of errors (touching boxes that have been found to be empty or revisiting boxes that have already been found to contain another token). (E) Paired Associates Learning (PAL). Boxes displayed on the screen were opened in a randomised order. Two or more of them contained a pattern. The patterns were then displayed on the screen, one at a time, and the child had to touch the box where the pattern was originally located. If the child made an error, the boxes were opened again, one at a time, to remind the child of their location. (F) Simple Reaction Time (RTI). The child had to react as soon as the yellow dot appeared on the screen by letting go of the press-pad button and pressing the screen where the dot appeared.

To obtain a measure of fine motor skill performance, the children were exposed to a visuomotor accuracy-tracking (VAT) task on test day 2. The task was a modified version of the VAT previously applied by Roig et al. [17] and Thomas et al. [18] using a customised software application (Matlab R2013b, Mathworks) and was chosen because it requires highly accurate hand movements based on visual inputs. In short, the children were comfortably seated, with their forearm resting on a table. In front of them was a 19-in computer screen, which was placed 25 cm from the edge of table. A cursor drawing a trace moved automatically across the screen from left to right in 8 s at a constant horizontal velocity and the children controlled the vertical position of the trace by moving a computer mouse. For each trial, the children were required to match the cursor with a fixed target as accurately as possible (Fig 1A) using their preferred hand to control the computer mouse. Following each trial, there was a 2-s pause. After a careful verbal instruction, demonstration and three practice trials without the target, the child completed 10 trials. No augmented feedback on performance was provided during the test. Offline, a score of 0‒100 was calculated for each trial based on the root mean square (RMS) error (distance) from the target, where 100 represented no RMS error and 0 represented a mean error > 2*target mean. The average score for the 10 trials was used as VAT performance.

#### Gross motor skills–coordination wall

To evaluate gross motor skill, each child completed a three-stage coordination wall task, which was previously used to test gross motor coordination in 10–13 year old children [19]. For each stage, the child stood in front of a table consisting of 9x8 A5-size squares with numbered marks from 1 to 10 distributed on the grid (Fig 1B). Half the numbers were blue, half red. The grid was also divided into an upper (top seven rows) and a lower section (bottom two rows) by a thick black line. The children had a red dot on their right hand and foot and a blue dot on their left hand and foot, and were instructed to touch the numbers, as fast as possible, from one to ten in the correct order with the hand (above the black line) or foot (below) corresponding to the colour of the number. If they made a mistake, they were told to correct the mistake and proceed. From stage 1 to 3, the task became more challenging. Stage 1 did not require crossing over the vertical midline (red numbers were on the right side of the grid and blue numbers on the left), while in stage 2 the red numbers were on the left side of the grid and blue numbers on the right, so that the child had to cross over the vertical midline to touch the numbers with the correct hand/foot. In stage 3, both red and blue numbers appeared on both sides (as depicted in Fig 1B).

Following introduction to the task, each child was given three attempts at each stage and the best (shortest) completion times (in s) from each stage were summed to get a combined score for each child.

#### Exercise capacity—Yo-Yo intermittent recovery level 1 children's test (YYIR1C)

A modified version of the Yo-Yo intermittent recovery level 1 (YYIR1C) [20] was used to measure the children’s fitness levels. It was recently shown that the YYIR1C test is reliable and a valid indicator of aerobic fitness in children younger than 10 years [20]. The test was carried out in the schools’ own settings. After a standardised warm-up procedure, including the first three running bouts, the children had to run 16 m back and forth in a straight lane marked by cones followed by a 10-s recovery period in which they walked around a cone 4 m behind the running lane. The running pace increased throughout the test and was determined by beeps from loudspeakers. The first time a child did not finish the 2x16 m in time, a warning was given. The second time, the test was over and the last level completed in time was noted by scientific personnel and the number of metres run was calculated. Scientific personnel also participated in the test to ensure that the children knew the right pace and to motivate the last one running.

### Cognitive Functions

The children were exposed to several standardised tests to assess performance and evaluate different domains of cognitive functions including simple reaction time, sustained attention ability, spatial working memory, the ability to learn paired associates and free-recall word memory. All cognitive tests were administered individually for each child. The child was placed sitting in front of a computer monitor and introduced to each task by an experimenter. Three children were tested simultaneously with no interaction between children.

In order to test different cognitive domains objectively, we used tests from the Cambridge Neuropsychological Test Automated Battery (CANTAB) supplemented by a free-recall word memory test. The CANTAB tests have been used in previous studies to evaluate cognitive functions also in children from 4–12 years [21]. The children were seated in front of a 23-in. touchscreen computer placed 30 cm from the edge of the table and with a press-pad with the response button located 15 cm from the screen. In addition to a motor screening task (MOT), which was used to introduce the touchscreen and software layout to the child, the CANTAB measurements included the following tests:

#### Reaction time

In this test named RTI (CANTAB), the child had to react as soon as a yellow dot appeared on the screen by letting go of the press-pad button and pressing the screen where the dot appeared (simple reaction time–child mode). After five practice runs, the child performed 15 test runs. The outcome measure was the average time (ms) from when the dot appeared to when the child let go of the button (Fig 1F).

#### Sustained attention ability

The test named Rapid Visual Processing (RVP, CANTAB) measured the ability of the child to sustain visual attention. A white box appeared in the centre of the computer screen, inside which digits, from 2 to 9, appeared in a pseudo-random order at the rate of 100 digits per minute. The child had to detect the target sequence (3-5-7) and to register responses using the press-pad (Fig 1C). The outcome measure used here was the number of missed target sequences. We used the "3-5-7 mode" recommended for children aged 7‒14 [22].

#### Spatial working memory

The spatial working memory test (SWM, CANTAB) requires retention and manipulation of visuospatial information and the test is employed to obtain a measure of executive function. In the test, the child had to search for a blue token hidden inside one of the coloured boxes shown on the screen (Fig 1D). The number of boxes was gradually increased from three to eight. The outcome measure used here was total number of errors (touching boxes that had been found to be empty or revisiting boxes that had already been found to contain a token). The test mode used was "shortened-3X3p-2X4-40-2X6-60-2X8-80".

#### Paired associates learning

The paired associates learning test (PAL, CANTAB) assesses visual memory and new learning abilities. Boxes displayed on the screen, in which two or more of them contained a pattern, were opened in a randomised order. The patterns were then displayed on the screen, one at a time, and the child had to touch the box where the pattern was originally located (Fig 1E). If the child made an error, the boxes were opened again, one at a time, to remind the child of their location. The number of patterns increased from two to eight. The total number of errors was used as the outcome measure. The “parallel_pa2_sixAttempts3” mode was used.

For a detailed description of CANTAB, see http://www.cambridgecognition.com.

#### Free-recall word memory

A word memory test similar to Pesce et al. [23] was used to evaluate free-recall semantic memory performance, since semantic memory is fundamental and could relate to scholastic learning. A 20-item word list was created by selecting highly concrete and imaginable nouns from the normative list of Paivio et al. [24]. The words were presented one at a time on a 15-in. computer screen for 5 s each using a recorded Microsoft PowerPoint presentation. After all 20 words had been presented, the children were instructed to close their eyes for 2 min (consolidation period), after which they were given 2 min to write down on a piece of paper all the words they remembered, in no particular order. The children were told that correct spelling was irrelevant and that we would ask them if a written word was not understood (this was rarely necessary).

### Academic Performance

To assess academic performance, the study focused on assessing the children’s abilities in mathematics and reading comprehension. The children were asked to perform standardised tests from Hogrefe Psykologisk Forlag A/S administered by the experimenters in the morning during school hours.

#### Mathematics

Proficiency in mathematics was assessed using a Danish standard, diagnostic, electronic test designed to measure mathematics skills relative to 3^rd^ grade level (MG3). The test included 50 problems relating to arithmetic's (addition, subtraction and multiplication), geometry and probability [25]. The children were seated in front of a PC, instructed to solve all 50 problems to the best of their abilities, and given all the time they needed to complete the test (no child spent more than 90 min). Three instructors were present to answer questions related to understanding of the problems but were not allowed to help with solving them. The outcome measure was number of correctly solved problems.

#### Reading comprehension

Reading performance was assessed using a Danish standardised reading comprehension test (Sætningslæseprøve 2) with good reliability and validity [26]. The test is designed to be used in 2^nd^ to 5^th^ grade and consists of 27 drawings of a situation, each accompanied by four sentences formulated as statements (108 problems in total) [26]. For each sentence, the child had to evaluate whether the statement matched the situation in the drawing by ticking a true or false box. One example was a drawing of a man sitting in a chair by a table in the living room drinking a cup of coffee. The four statements accompanying this drawing were: 1) Next to the cup was a blue coffee pot. 2) The man had gotten a bottle and a can. 3) He sat in his back yard and drank coffee. 4) Every morning he drank coffee with his wife. In this example, only the first statement matched the drawing, but all four statements could be true or false for other drawings. The sentences gradually increased in length and complexity. The child was given 8 min to evaluate as many statements as possible. The outcome measure was number of correctly evaluated statements.

### Statistics

For the statistical analyses in this cross-sectional study we used R (R Core Team, 2015). Characteristics and test performances of the study population were summarized, using mean and standard deviations for continuous variables and counts per group for categorical variables. Pairwise associations between the above-mentioned measures of cognitive function, physical capacity and motor skills and academic performance were investigated by means of linear mixed-effects models, which assumed a linear relationship between the two correlates (x and y) while allowing adjustment for imbalances in age, sex, Tanner stage, municipality, and school classes (modelled using random effects), across the range of the values of the correlate on the x axis [27, 28]. For the tests of associations between motor skills, exercise capacity and cognitive function, the measures of cognitive function were treated as y variables whereas measures of motor skills and physical capacity were treated as x variables. Estimated slope coefficients quantifying the increase in the y variable (e.g., additional number of errors or words) for each unit increase in the x variable with corresponding standard errors were reported; these estimates allowed direct interpretations as conversion factors between measures of motor skills and physical capacity and measures of cognitive function and academic performance. Approximate t-tests were used to evaluate the strength of the associations. Model checking was carried out by visual inspection of residual plots and normal probability plots.

## Results

The characteristics of the children included in the study as well as their physical, cognitive and academic performance outcome measures are presented in Table 1.

### Cross-Sectional Associations between Motor Skills, Exercise Capacity and Cognitive Functions

Table 2 shows the cross-sectional associations between motor skills, exercise capacity and performance in different cognitive domains. Performance in both the fine and gross motor skill task was significantly associated with better performance in each of the cognitive tests (all P<0.001; except for the association between gross motor skills and PAL where P = 0.013). Participation in organised leisure sports was associated with better semantic memory (0.58±0.03, P = 0.025), but with no other cognitive domains. Higher exercise capacity was associated with fewer errors in the spatial working memory (-0.30±0.10, P = 0.038) and sustained attention (-0.11±0.04, P = 0.046) tests. There were no significant associations between fat% and performance in any of the tested cognitive domains.

**Table 2:** Cross-sectional associations between motor skills, exercise capacity and measures of cognitive functions.

	Simple reaction time (ms)		Sustained attention (RVP #misses)		Spatial working memory (#errors)		Free-recall word memory (#words)		Paired associates learning (#errors)	
	Estimate (SE)	P-value	Estimate (SE)	P-value	Estimate (SE)	P-value	Estimate (SE)	P-value	Estimate (SE)	P-value
***Motor skills***										
Fine motor skill (VAT score)	**-1.10 (0.24)**	<0.001	**-0.07 (0.01)**	<0.001	**-0.13 (0.03)**	<0.001	**0.03 (0.01)**	<0.001	**-0.24 (0.05)**	<0.001
Gross motor skill (motor-skill wall, s)	**1.03 (0.22)**	<0.001	**0.08 (0.01)**	<0.001	**0.18 (0.03)**	<0.001	**-0.04 (0.01)**	<0.001	**0.13 (0.04)**	0.013
***Exercise capacity*, *leisure sport and body fat***										
Exercise capacity (YYIR1C per 100 m)	-1.92 (0.81)	0.103	**-0.11 (0.04)**	0.046	**-0.30 (0.10)**	0.038	0.03 (0.02)	0.628	0.18 (0.16)	0.828
Organised leisure sports (if yes)	-9.71 (7.15)	0.677	-0.31 (0.38)	0.958	-0.82 (0.92)	0.937	**0.58 (0.03)**	0.025	-0.94 (1.36)	0.981
Body fat (%)	0.15 (0.41)	0.999	0.02 (0.02)	0.858	0.13 (0.05)	0.091	-0.01 (0.01)	0.997	0.03 (0.08)	0.998

Associations with a significant estimate are shown in bold. The estimates represent slope coefficients quantifying the increase in the y variable (e.g., additional number of errors in the spatial working memory task) for each unit increase in the x variable (e.g. for each extra second spent to complete the gross motor skill task).

### Cross-Sectional Associations–Predictors of Academic Performance

Cross-sectional associations between academic performance and measures of cognitive functions, motor skills and exercise capacity, respectively, are shown in Table 3. All measures of performance in cognitive tests were associated with better academic performance in both mathematics and reading comprehension. Measures of both fine and gross motor skill as well as exercise capacity were also associated with better academic performance. Participation in organised leisure sports significantly predicted reading (4.30±1.59, P = 0.040) but not mathematics performance, although a tendency was evident (2.19±0.87, P = 0.067). There was no significant association between body fat% and academic performance. The associations between performance in cognitive tests, motor performance and academic performance are described in further detail below.

**Table 3:** Cross-sectional associations between measures of cognitive functions, motor skills and exercise capacity respectively, and academic performance.

	Mathematics (#correct)		Reading comprehension (#correct)	
	Estimate (SE)	P-value	Estimate (SE)	P-value
***Performance in cognitive tests***				
Simple reaction time (per 10 ms)	**-0.21 (0.06)**	0.002	**-0.47 (0.11)**	<0.001
Sustained attention (RVP #misses)	**-0.78 (0.11)**	<0.001	**-1.18 (0.20)**	<0.001
Spatial working memory (SWM #errors)	**-0.28 (0.05)**	<0.001	**-0.28 (0.09)**	0.006
Free-recall word memory (#words)	**1.70 (0.20)**	<0.001	**3.09 (0.37)**	<0.001
Paired associates learning (PAL #errors)	**-0.15 (0.03)**	<0.001	**-0.23 (0.06)**	<0.001
***Motor skills***				
Fine motor skill (VAT score)	**0.20 (0.03)**	<0.001	**0.26 (0.05)**	<0.001
Gross motor skill (motor-skill wall, s)	**-0.22 (0.03)**	<0.001	**-0.32 (0.05)**	<0.001
***Exercise capacity*, *leisure sport and body fat***				
Exercise capacity (YYIR1C –per 100 m)	**0.40 (0.10)**	<0.001	**0.51 (0.18)**	0.029
Organised leisure sports (if yes)	2.19 (0.87)	0.067	**4.30 (1.59)**	0.040
Body fat (%)	-0.12 (0.05)	0.103	0.08 (0.09)	0.950

Associations with a significant estimate are shown in bold. The estimates represent slope coefficients quantifying the increase in the y variable (e.g., additional number of correct answers in the mathematics test) for each unit increase in the x variable (e.g. for each extra VAT score point in the fine motor skill task).

### Performance in Cognitive Tests Correlates with Academic Achievement

**Fig 2:**
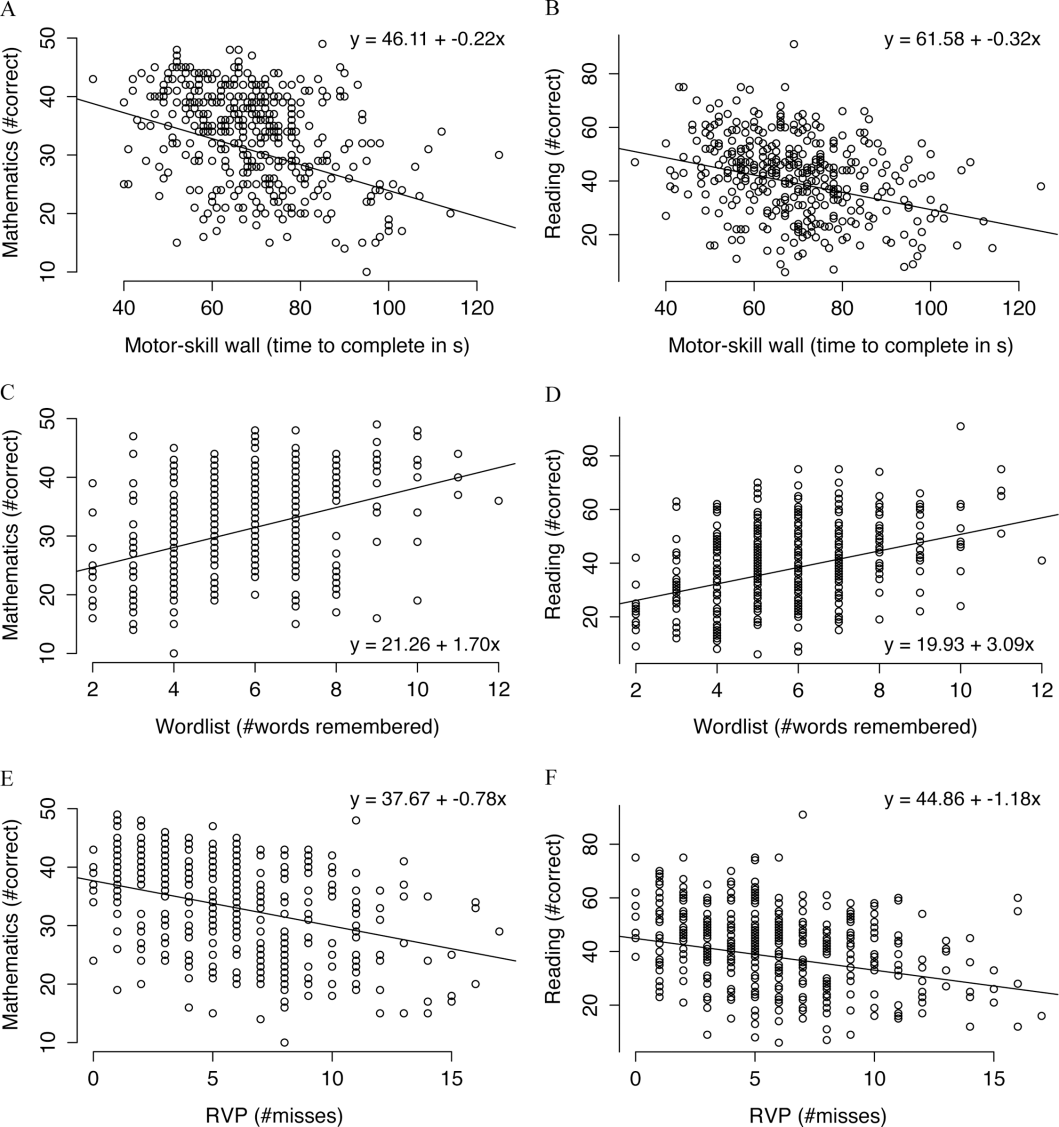
Performance in gross motor skill, memory and attention tests plotted against academic performance. (A and B) Time to complete the motor-skill wall and mathematics and reading performance. (C and D) Number of words remembered from the 20-item word list in the free-recall word memory task and mathematics and reading performance. (E and F) Number of missed sequences in the RVP sustained attention task and mathematics and reading performance. The equation for the regression line is inserted in each plot.

Performance in all cognitive tests correlated with academic performance. Simple reaction time, a measure of processing speed, was associated with better academic performance (mathematics: -0.21±0.06, P = 0.002; reading: -0.47±0.11, P<0.001). Better attention, as measured by the number of misses in the RVP test, was associated with better performance in both mathematics (-0.78±0.11, P<0.001, Fig 2E) and reading (-1.18±0.20, P<0.001, Fig 2F). Significant associations with academic performance were also observed for spatial working memory (mathematics: -0.28±0.05, P<0.001; reading: -0.28±0.09, P = 0.006) and for the ability to remember words (mathematics: 1.70±0.20, P<0.001, Fig 2C; reading: 3.09±0.37, P<0.001, Fig 2D). Finally, the ability to learn paired associates was also associated with better test performance in mathematics (-0.15±0.03, P<0.001) and reading (-0.23±0.06, P<0.001).

#### Motor skills and exercise capacity predict academic achievement

Fine motor skill (VAT score) was associated with better academic performance, with estimates of 0.20±0.03 (P<0.001) and 0.26±0.05 (P<0.001) for mathematics and reading, respectively. Better performance in gross motor skill (i.e. shorter time to complete the wall) was also associated with better scores in both the mathematics (-0.22±0.03, P<0.001, Fig 2A) and the reading test (-0.32±0.05, P<0.001, Fig 2B). In regard to exercise capacity, the statistical tests showed that for each 100 m run in YYIR1C, the children scored 0.40±0.10 higher in the mathematics test (P<0.001) and 0.51±0.18 higher in the reading comprehension test (P = 0.029).

## Discussion

This study is the first to show consistent cross-sectional associations between performance in tests of fine and gross motor skills and objectively measured performance in a wide range of cognitive domains in a large sample of preadolescent children. Furthermore, the results demonstrate that performance in tests of motor skills, exercise capacity and cognitive functions correlates with academic performance in tests of both mathematics and reading comprehension in this population of children.

### Fine and Gross Motor Skills Correlate with Performance in Cognitive Tests

We often consider the development of motor skills as separate from cognitive development, and the terminology itself clearly separates these functions. The present results do, however, demonstrate a clear association between both gross and fine motor skills and performance in cognitive domains such as spatial working memory, wordlist memory (semantics), sustained attention, reaction time (speed) and the ability to learn paired associates. Although the findings are observational and the interrelationship between motor and cognitive skills is naturally complex, the present results emphasise the importance of focusing on this particular association.

Recent studies have focused on the association between aerobic fitness and cognitive functions in preadolescent children, adolescents and adults [1, 4, 29–32]. The present results confirm that exercise capacity or aerobic fitness is positively associated with performance in certain cognitive domains such as spatial working memory and sustained attention. Our results also demonstrate associations between participation in organised leisure sport and, for example, free-recall word memory performance. These findings indicate a positive relation between physical activity levels and specific cognitive functions, whereas body composition did not correlate with performance in any cognitive tests.

It is, however, important to emphasise the close associations between motor skills and performance in cognitive tests compared to exercise capacity. Both gross and fine motor skills were also positively associated with performance in a wider range of cognitive domains, including simple reaction time (speed) and the ability to learn paired associates. Most public physical activity guidelines focus solely on quantity and intensity of physical activity and suggest aerobic and strengthening activities [33, 34], but the present results indicate that it is important to also pay specific attention to motor skills. School PE provides an obvious opportunity to prioritise also training of motor skills, as performance in motor skill tests also relate to academic achievement.

### Performance in Cognitive Tests Correlate with Academic Achievement

Academic performance was assessed as reading comprehension and the ability to solve mathematical problems in standardised, validated tests. We found that performance in all cognitive domains was positively associated with both mathematics and reading performance. Thus, spatial working memory and wordlist memory, sustained attention ability, reaction time (speed) and the ability to learn paired associates correlated with both mathematics and reading performance. This result fits well with previous studies demonstrating that, for example, attention is a strong predictor of later mathematics and reading scores [35]. Reading comprehension test performance draws on the long-term and working memory, and reflects the reading comprehension of the child, which includes accurate and fluent decoding of words and vocabulary knowledge (i.e. semantic memory), whereas mathematics requires knowledge of numbers, arithmetic concepts and geometry. Both reading comprehension and mathematics require problem solving, logical thinking and reasoning.

### Fine and Gross Motor Skills Correlate with Academic Achievement

Importantly, both fine and gross motor skills were also positively associated with both mathematics and reading performance. Thus, shorter time to complete the gross motor skill test and higher accuracy in the fine motor skill test correlated with academic performance. Although the mechanisms underlying the association between motor skills and academic performance remain unclear, the strengths of these associations are noteworthy. In addition to fast and accurate movements (i.e. motor control), the motor skill tests also engage cognitive processes, including decision-making, sustained attention and processing speed. Thus, motor skill practice may promote academic performance through an effect on cognitive resources. Furthermore, it may be speculated that both reading and the ability to solve mathematical problems also involve elements of skill learning and that a positive role of meta-learning is involved. Indeed, recent studies have provided important knowledge on the interrelationship between childhood motor functions and adolescent academic performance [12, 35]. Even more importantly, Ericsson & Karlsson [11] recently demonstrated in a longitudinal study that daily PE and increased focus on motor skill training during compulsory school years can improve both motor skills and academic performance in adolescence. Again, this emphasises the potential and importance of focusing on motor skills in schoolchildren.

As shown in previous studies, we found significant associations between aerobic fitness and academic performance in mathematics and reading [4, 9, 10], but we also showed that participation in organised leisure sports significantly correlated with reading performance (and tended to correlate with mathematics performance). Whether these associations are related to the actual aerobic fitness of the child or mediated via other mechanisms is impossible to determine. Higher exercise capacity has been associated with better cognitive function in children [30, 31], and interventions that improve aerobic fitness may also lead to improved cognitive functions [32]–possibly through plastic changes in the central nervous system [36, 37]. In the present study, the association with academic performance may, however, also be mediated by general physical activity levels and motor skills. Kantomaa et al. [12] recently found that physical activity mediates the association between childhood motor functions and adolescent academic performance. This finding is noteworthy and emphasises the importance of general physical activity. However, it does not resolve the relative contribution of exercise capacity in itself. Thus, higher general physical (motor) activity may lead to improved exercise capacity in the active children, but it could also lead to improved motor functions and thus indirectly mediate the association between aerobic fitness and academic performance.

### Limitations

Although we adjust for age, sex, Tanner stage, municipality, and school class in our models, some residual potential confounding cannot be ruled out. In particular it would have been preferable to be able to adjust for intelligence quotient, socioeconomic status and parental education. Nevertheless, the consistency of our findings with similar trends across a wide range of different performance measures indicates some degree of robustness in our results.

## Conclusions

The data demonstrate a significant positive correlation between children’s fine and gross motor skill levels and performance in all employed cognitive tests. Exercise capacity (aerobic fitness) was also associated with performance in some aspects of cognitive functioning (e.g. spatial working memory). Moreover, the results demonstrate that performance in tests of motor skills, exercise capacity and particular aspects of cognitive functions, including sustained attention, spatial working memory, episodic and semantic memory, and processing speed, are significantly correlated with academic performance in tests of both mathematics and reading comprehension.

The fact that motor skills were positively correlated to performance in all domains of both cognitive and academic tests suggest that it is important to focus on general motor skill development in schoolchildren. Future intervention studies focusing on motor skill training should investigate how improvements in fine and gross motor skills affect cognitive functions and academic performance in children and the possible underlying mechanisms should be investigated e.g. by using neuroimaging techniques.
